# Microvascular invasion and early recurrence of hepatocellular carcinoma after CT-guided radiofrequency ablation: risk factor analysis

**DOI:** 10.3389/fonc.2025.1672300

**Published:** 2025-10-21

**Authors:** Yuyan Liu, Xiaoyang Zhao, Lupeng Li, Huicun Cao

**Affiliations:** Department of Intervention, Henan Provincial People’s Hospital, People’s Hospital of Zhengzhou University, Zhengzhou, Henan, China

**Keywords:** hepatocellular carcinoma, CT-guided radiofrequency ablation, microvascular invasion, early recurrence, risk factors

## Abstract

**Background:**

Hepatocellular carcinoma (HCC) remains a major global health challenge, and microvascular invasion (MVI) and early recurrence pose significant obstacles to effective treatment. Identifying the risk factors associated with these complications following computed tomography (CT)-guided radiofrequency ablation (RFA) is essential for optimizing patient management and improving treatment outcomes.

**Methods:**

A retrospective analysis was conducted from January 2020 to January 2022, involving 186 patients who underwent CT-guided RFA for primary HCC at a single institution. The study assessed tumor characteristics, liver function, and post-treatment outcomes to identify predictors of MVI and early recurrence. Logistic regression and machine learning were employed to determine statistically significant risk factors.

**Results:**

Multiple tumors, incomplete capsules, irregular tumor margins, and rapid portal venous washout were identified as significant predictors of MVI. Similarly, rapid portal venous phase washout, tumor internal necrosis, MVI, multiple tumors, and incomplete capsule integrity were strongly associated with early recurrence. The results of the logistic regression machine learning further enhance the reliability of the current findings.

**Conclusion:**

Patients with HCC exhibiting certain high-risk features are susceptible to MVI and early recurrence following CT-guided RFA. The identified risk factors suggest the need for enhanced monitoring and personalized therapeutic strategies to improve patient outcomes.

## Highlights

- Significant Predictors Identified: Multiple tumors, incomplete capsules, and irregular tumor margins significantly predict microvascular invasion in HCC post-RFA.
- Early Recurrence Correlation: Features such as rapid portal venous phase washout and tumor internal necrosis are closely linked with early recurrence of HCC.
- Stratification of Risk: The study effectively stratifies risk levels in HCC patients undergoing RFA based on specific tumor and patient characteristics.
- Implications for Monitoring: Highlighting these risk factors emphasizes the need for intensified monitoring and tailored therapeutic strategies for high-risk patients.
- Enhancing Treatment Outcomes: By understanding these risk factors, clinicians can better predict and manage potential complications, potentially enhancing overall treatment outcomes.

## Introduction

Primary hepatocellular carcinoma (HCC) represents a major global health burden due to its increasing incidence and high mortality rates. PHC is mainly diagnosed at an advanced stage, limiting curative treatment options and highlighting the need for effective early interventions (1–3). Computed tomography (CT)-guided radiofrequency ablation (RFA) has emerged as a promising therapeutic approach for small to medium-sized tumors. This technique enables precise tumor cell destruction while maintaining hepatic function and enhancing prognostic outcomes (4, 5).

However, the effectiveness of RFA can be significantly compromised by microvascular invasion (MVI) and early recurrence of the disease (6, 7). The presence of MVI not only facilitates the spread of cancer cells through blood vessels, but also poses challenges in achieving complete ablation, thereby increasing the risk of early recurrence (8, 9). Early recurrence, defined as the reappearance of HCC within two years post-treatment, significantly reduces patients’ long-term survival and quality of life. Identifying the risk factors for MVI and early recurrence in PHC patients undergoing CT-guided RFA is crucial for improving treatment outcomes. Several studies have indicated that variables, such as tumor size, location, and the presence of satellite nodules, significantly influence the risk of MVI and subsequent recurrence (10).

The present study aimed to systematically analyze the risk factors for MVI and early recurrence following CT-guided RFA in PRIMARY HCC patients. By identifying reliable predictive factors, the findings may help optimize individualized treatment strategies, improving therapeutic outcomes and patient survival.

## Methods

### Study design

A retrospective analysis was conducted at Henan Provincial People’s Hospital to investigate the risk factors for MVI and early recurrence following CT-guided RFA in PHC patients. The study covered cases from January 2020 to January 2022. A total of 186 patients who underwent CT-guided RFA were included in the analysis. Ethical approval was obtained from ethics committee of Henan Provincial People’s Hospital (2022-75), and informed consent was signed by all participants.

## Case selection criteria and study factor determination process

### Case inclusion criteria

1. Tumor Characteristics: Patients with a single tumor with a maximum diameter of ≤5 cm or up to three tumors, each ≤3 cm in diameter.
2. Vascular and Organ Invasion: No evidence of vascular invasion (macrovascular invasion), invasion of adjacent organs, or extrahepatic metastases.
3. Liver Function: Liver function was assessed using albumin and bilirubin levels as well as Child-Pugh classification. Baseline liver function characteristics are as follows: mean albumin (3.5 g/dL), mean bilirubin (1.2 mg/dL), and distribution of Child-Pugh scores. The sample included a mix of well-preserved and compromised liver function, providing a representative analysis across the spectrum of cirrhotic liver states.
4. Consent: All participants must provide voluntary, written informed consent to enroll in the study.

### Case exclusion criteria

1. Recent Hemorrhage: Patients with a history of esophageal or gastric fundal variceal bleeding within one month prior to treatment were excluded.
2. Coagulopathy and Hematologic Diseases: Patients with uncorrectable coagulation disorders, severe blood dyscrasias, or a significant bleeding tendency were regarded ineligible.
3. Ascites and Cachexia: Patients with refractory, extensive ascites or cachexia were excluded.
4. Active Infection: Patients with active infections, especially biliary system infections, were regarded ineligible.
5. Severe Organ Dysfunction: Patients with severe dysfunction of major organs, such as the liver, kidneys, heart, lungs, or brain, were excluded.

Patients with MVI were excluded from this study, as indicated by the case selection criteria, which required the absence of vascular invasion, invasion of adjacent organs, or extrahepatic metastases.

Etiology of cirrhosis was assessed and classified as hepatitis B virus (HBV), hepatitis C virus (HCV), non-alcoholic fatty liver disease (NAFLD), and others, with the following distribution: HBV (45%), HCV (30%), NAFLD (15%), and other causes (10%). The incidence of microvascular invasion (MVI) varies significantly across these etiological subgroups, and subsequent analyses will stratify findings based on etiology to better define the risk profile for MVI in these distinct groups.

### Study factor determination process

The determination of predictive factors in this study followed evidence-based medical methodologies, integrating empirical evidence, biological mechanisms, clinical applicability, and expert consensus to ensure both model accuracy and clinical utility. A systematic literature review was first conducted to analyze known biological mechanisms of HCC recurrence and microvascular invasion post-RFA, identifying imaging and clinical characteristics closely associated with tumor aggressiveness. Candidate factors underwent multidimensional validation, including assessments of clinical relevance and confirmation of pathophysiological rationality. A multidisciplinary panel comprising hepatologists, interventional radiologists, and surgeons further refined the selection through consensus, prioritizing predictive factors that directly influence clinical decision-making. Finally, statistical considerations were applied to balance predictive power with practical implementation, eliminating redundant variables and focusing on measurable pre-procedural CT features. Factors assessed included Child-Pugh grade, tumor internal necrosis, arterial phase enhancement, number of tumors, capsule integrity, tumor margin, and portal venous phase washout.

### Procedure of tissue sample acquisition and RFA

This study employed a dual tissue acquisition approach combining percutaneous needle biopsy and radiofrequency ablation (RFA). The anesthesia protocol was tailored to individual cases, with most patients receiving local anesthesia combined with intravenous sedation and analgesia, while general anesthesia was administered for complex cases. The biopsy procedure utilized CT-guided 18G core needle sampling to obtain 2–3 tissue specimens for pathological analysis.

Equipment: The RFA procedures were performed using the Lide LDRF-120S radiofrequency ablation system. This device was designed to ensure precision and safety in the ablation of HCC lesions under imaging guidance.
RFA Procedure: The procedure commenced with accurate aseptic preparation and draping of the operative field. Utilizing real-time CT guidance, a unipolar RFA probe was advanced along a pre-determined trajectory, accompanying by incremental adjustment of insertion depth and angulation to ensure optimal alignment with the target ablation zone. Needle advancement was executed in a stepwise manner, incorporating intra-procedural imaging to refine positioning and maximize contact between the probe’s active tip and the tumor core. Once accurate placement was confirmed, the electrode was stabilized to mitigate the risk of displacement during the ablation process. Precise documentation of electrode trajectory, depth, and orientation was maintained throughout. Following completion of the ablation cycle, track ablation was systematically performed during probe withdrawal to minimize the likelihood of hemorrhage and tumor seeding along the needle path. A comprehensive post-ablation CT scan was conducted to verify the extent of thermal necrosis, ensuring complete tumor coverage with a circumferential safety margin of 0.5–1.0 cm. This final imaging assessment was instrumental in evaluating treatment efficacy and identifying potential complications, such as tumor rupture, vascular injury, or pneumothorax. Post-procedural management encompassed hepatoprotective interventions and multimodal analgesia, facilitating optimal recovery while mitigating procedure-related morbidity. Given the potential for underrepresentation of MVI in ultrasound-guided biopsies, caution is advised when interpreting these biopsy results, as they may not adequately reflect the true MVI status observed with CT guidance.

### MVI histopathological assessment protocol

The criteria for determining MVI involve meticulous postoperative pathological examinations. Under microscopic analysis, MVI is identified by the presence of clusters of cancer cells in the lumens of endothelium-lined blood vessels. This examination specifically concentrated on the veins and vascular structures in the peritumoral portal tracts and tumor capsule. The evaluation of MVI included studying the number and distribution of these microscopic invasions. On the following protocol, patients were classified into two groups: MVI-positive group and non-MVI group. Pre-treatment biopsy specimens were obtained under ultrasound guidance. However, the ultrasound-guided approach may not accurately localize the ‘rapid portal venous phase washout area’ as identified on CT imaging. This discrepancy may result in biopsy specimens that fail to capture MVI-positive tissue, potentially underestimating the incidence of MVI.

### MVI evaluation

Microvascular invasion was rigorously diagnosed according to the American Association for the Study of Liver Diseases Guidelines (AASLD) 2018 (11). The diagnostic criteria required:

Morphological confirmation: Tumor cell emboli within endothelial-lined vessels (portal veins ≥50 μm diameter, hepatic veins, or lymphatic channels) identified on hematoxylin-eosin (HE) stained sections. Capsular vessels and tissue processing artifacts were excluded through serial section analysis.
Immunohistochemical validation: CD34/CD31 (vascular markers) and D2-40 (lymphatic marker) staining were routinely performed to confirm endothelial lining integrity. Ambiguous cases underwent elastic fiber staining (EVG) to detect vascular wall structures.
MVI grading was stratified as(Cases diagnosed as both M1 and M2 grades are defined as MVI-positive):M0: No identifiable invasion;M1: 1–5 tumor foci in peritumoral vessels (≤1 cm from tumor boundary);M2: >5 foci or any invasion beyond 1 cm margin.

### Specimen handling protocol

For surgical specimens:

Entire tumor capsule and surrounding 2 cm parenchyma were serially sectioned at 5 mm intervals.
All suspicious vessels were sampled with ≥3 parallel sections for evaluation.

For RFA cohort:

Pre-treatment core biopsies were obtained under ultrasound guidance (18G needle, 3 passes minimum).
Biopsy adequacy was confirmed by on-site cytopathologist (≥3 portal tracts per specimen).

While the biopsy was initially performed under ultrasound guidance, it is noteworthy that this method does not always coincide with the CT-guided identification of the high-risk portal venous phase washout regions, possibly leading to missed MVI detection.

### Quality control measures

Double-blind review: Two board-certified hepatopathologists independently scored all slides using standardized criteria (AASLD2018).
Discrepancy resolution: Cases with initial disagreement (Cohen’s kappa coefficient κ=0.79-0.83) underwent third-review by a senior pathologist with 15-year HCC specialization.
External validation: 10% random samples were re-evaluated by an external expert panel (concordance rate 92.6%).

Discrepancies arising from the use of ultrasound for pre-treatment biopsies, especially when targeting the portal venous phase washout regions, were considered during the review process to ensure MVI detection accuracy.

### Monitoring and assessment protocol for early recurrence

Assessment During Monitoring: Patients enrolled in the study underwent a rigorous follow-up for two years after the conclusion of interventional therapy. Follow-up was conducted monthly via telephone and outpatient visits to ensure continuous monitoring and support. The follow-up regimen included routine physical examinations to monitor the patients’ general health and detect any physical signs of tumor recurrence. For patients suspected of experiencing recurrence, further diagnostic assessments were conducted, including pathology tests to confirm the presence of new tumor growth. Every three months, patients underwent comprehensive imaging assessments with chest and abdominal CT scans. These regular imaging sessions were crucial for detecting any changes in the liver or the emergence of new lesions indicative of recurrent HCC.

### Criteria for determining early recurrence

Early recurrence of HCC was defined as tumor reappearance within two years post-treatment. This was determined through imaging studies, supplemented by confirmatory diagnostics, such as percutaneous biopsy or post-operative pathological examination following any re-intervention. The composite risk score incorporated:

Major determinants: M1–2 grade, poor differentiation (Edmondson-Steiner Grade III or IV hepatocellular carcinoma), satellitosis (≥2 nodules within 2 cm).
Supportive features: Capsular integrity (complete/infiltrative); Resection margin status (Microscopic tumor involvement at resection margin (ink-positive) versus R0 resection with >1 mm tumor-free margin); Proliferation index (Ki-67 proliferation index ≥30% quantified via whole-slide digital image analysis), etc.

### Statistical analysis

The statistical analysis of data was carried out using SPSS 27.0 software (IBM, Armonk, NY, USA). Initially, all data were categorized as either quantitative or categorical. Normality tests were utilized to determine the distribution patterns of the quantitative data. All statistical tests were two-tailed, and a p-value of less than 0.05 was statistically considered significant. To account for the significant variability in MVI risk across different etiologies of cirrhosis, stratified analyses were conducted on patients with HBV-related HCC, HCV-related HCC, and those with other etiologies, such as NAFLD. These subgroup analyses allow for more precise risk prediction and clinical applicability in each etiology-specific cohort.

### Univariate analysis

For variables following a normal distribution, inter-group differences were analyzed using independent sample t-test, in which results were expressed as mean ± standard deviation. Categorical variables were summarized as frequency and percentage, and Chi-square (χ²) test was employed to examine associations or independence among these variables (**Supplementary Tables 1**, **2**).

### Multivariate analysis

**Figure 1:**
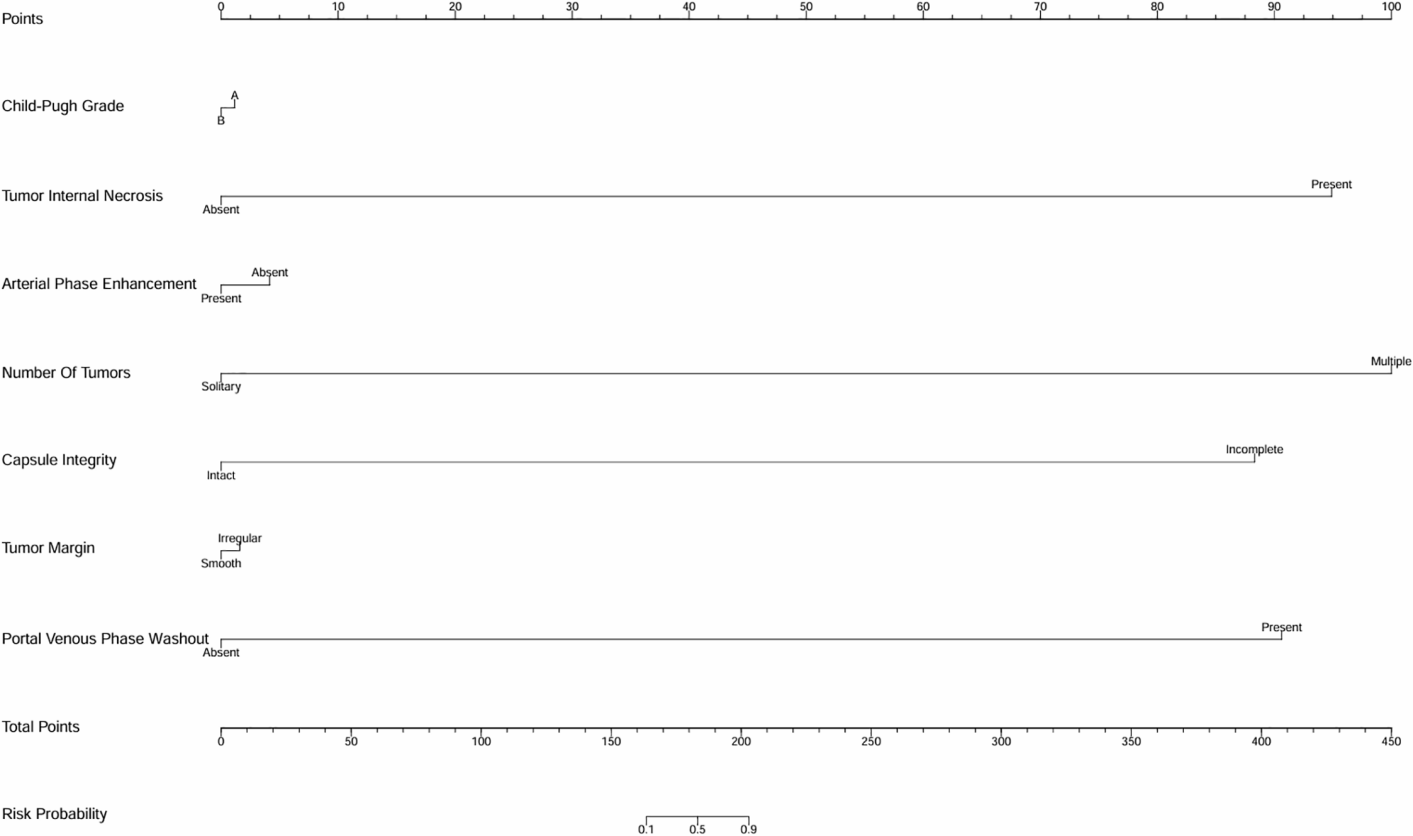
Univariate analysis nomogram for MVI risk prediction. The nomogram incorporates variables such as Child-Pugh grade, tumor internal necrosis, arterial phase enhancement, number of tumors, capsule integrity, tumor margin, and portal venous phase washout to estimate the probability of microvascular invasion (MVI).

**Figure 2:**
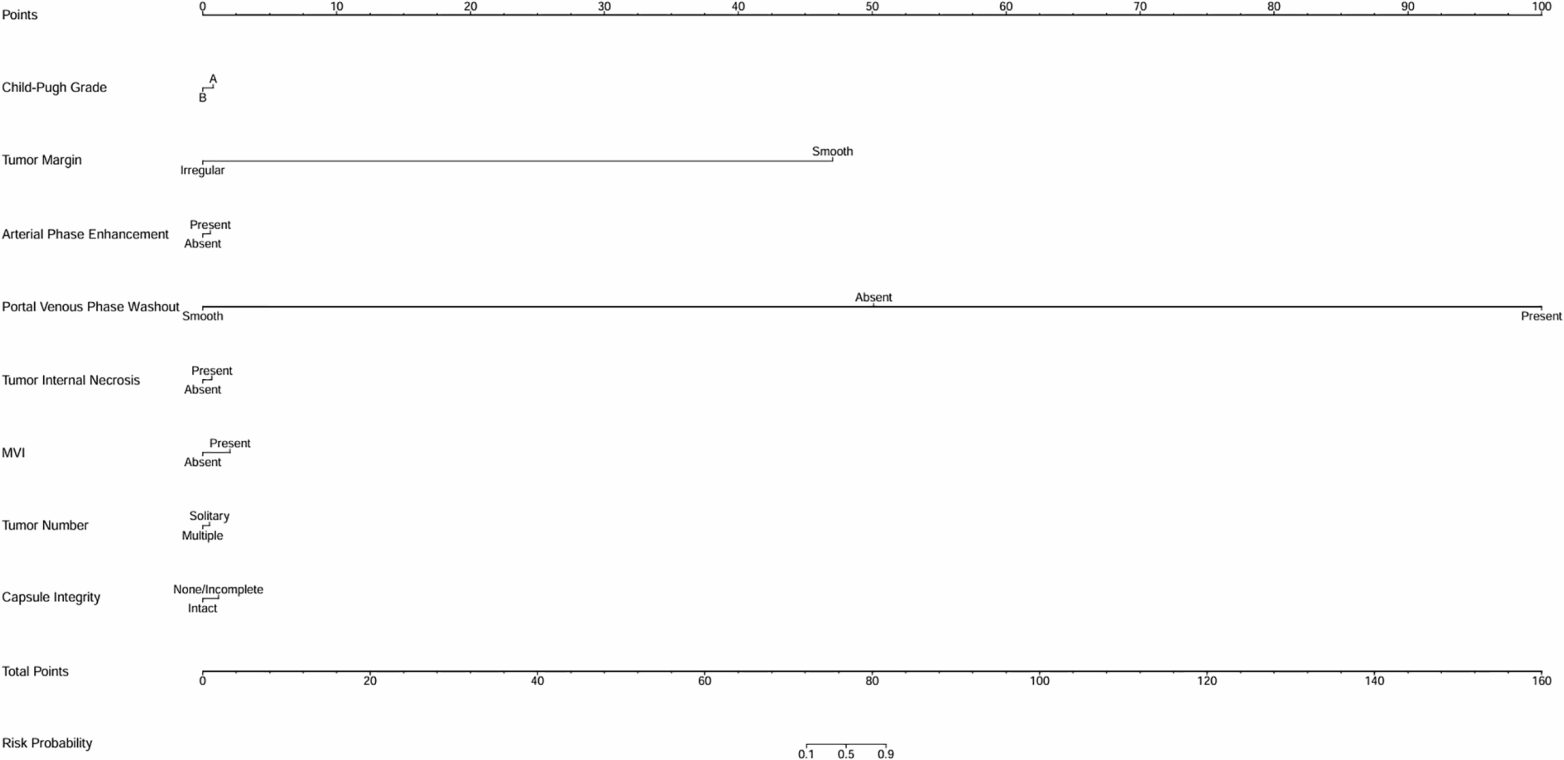
Univariate analysis nomogram for early recurrence risk prediction. This nomogram evaluates factors including tumor margin, arterial phase enhancement, portal venous phase washout, tumor internal necrosis, MVI status, tumor number, and capsule integrity to predict the likelihood of early recurrence.

Variables exhibiting significant effects in the univariate analysis were involved in the multivariate analysis. Logistic regression analysis was employed, providing odds ratios and confidence intervals to identify factors influencing outcomes. To demonstrate the discriminative ability and predictive performance of the logistic regression results, this study presents both the ROC curve (**Supplementary Figures 1**, **2**) and nomogram (**Figures 1**, **2**; **Table 1**) of the logistic regression model.

**Table 1:** Logistic regression analysis results.

Logistic regression analysis of risk factors for microvascular invasion in hepatocellular carcinoma.						
Factors (Abbreviations)	β value	Standard error	Wald value	OR (odds ratio)	95% CI (for OR)	p-value
Multiple Tumors	0.298	1.154	3.537	3.348	1.176 to 4.272	<0.05
Capsule Incomplete	0.225	0.991	2.554	2.498	1.135 to 3.720	<0.05
Irregular Tumor Margin	0.312	1.268	3.707	3.559	1.198 to 4.461	<0.05
Rapid Portal Venous Washout	0.265	0.922	3.157	2.982	1.069 to 3.967	<0.05

Logistic regression analysis of risk factors for early recurrence post-intervention in hepatocellular carcinoma.						
Factors (Abbreviations)	β value	Standard error	Wald value	OR (odds ratio)	95% CI (for OR)	p-value
Portal Venous Phase Washout	0.268	1.039	3.183	3.213	1.159 to 3.860	<0.05
Tumor Internal Necrosis	0.203	0.892	2.299	2.376	1.258 to 3.348	<0.05
Microvascular Invasion	0.281	1.141	3.337	3.324	1.078 to 4.246	<0.05
Multiple Tumors	0.239	0.830	2.843	2.893	0.982 to 3.601	<0.05
Incomplete Capsule	0.302	1.202	3.407	3.455	1.123 to 4.387	<0.05

### Machine learning of logistic regression model

**Figure 3:**
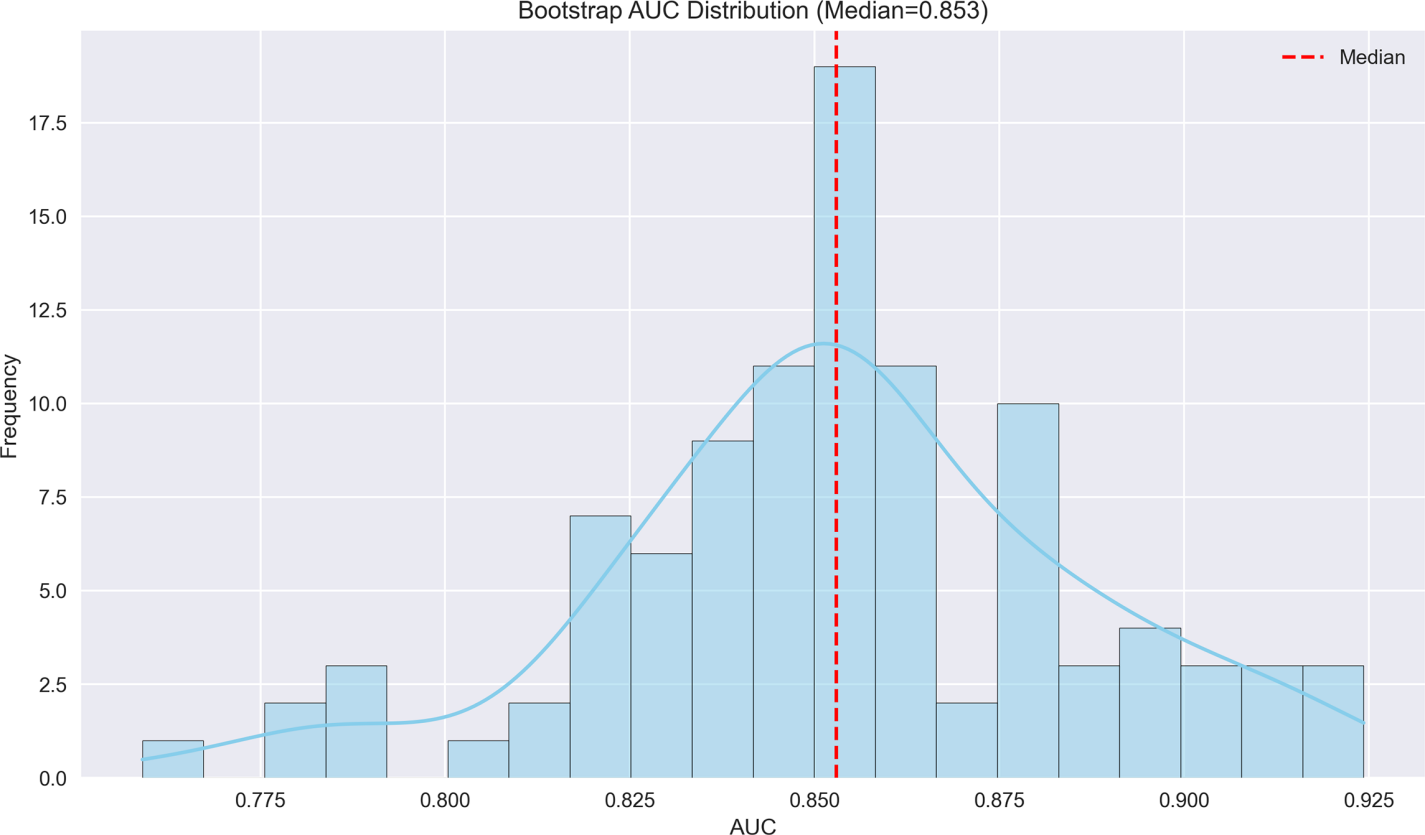
Bootstrap AUC distribution for MVI prediction model. The histogram displays the distribution of AUC values from bootstrap resampling, with a median AUC of 0.853, indicating robust discriminatory performance.

**Figure 4:**
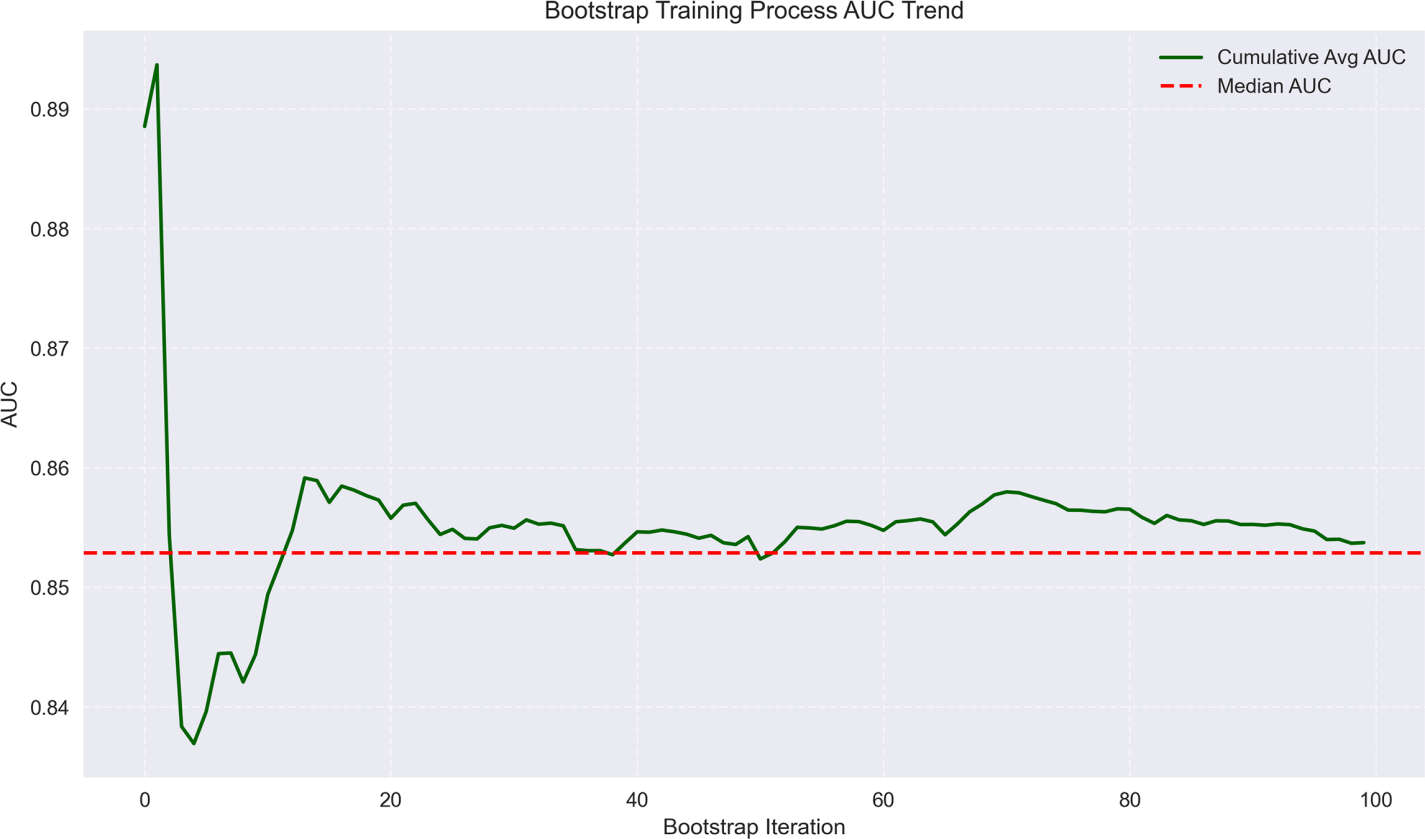
Bootstrap training process AUC trend for MVI prediction. The plot shows the trend of AUC values during bootstrap iterations, with cumulative average and median AUC stabilizing around 0.85–0.86, reflecting model consistency.

**Figure 5:**
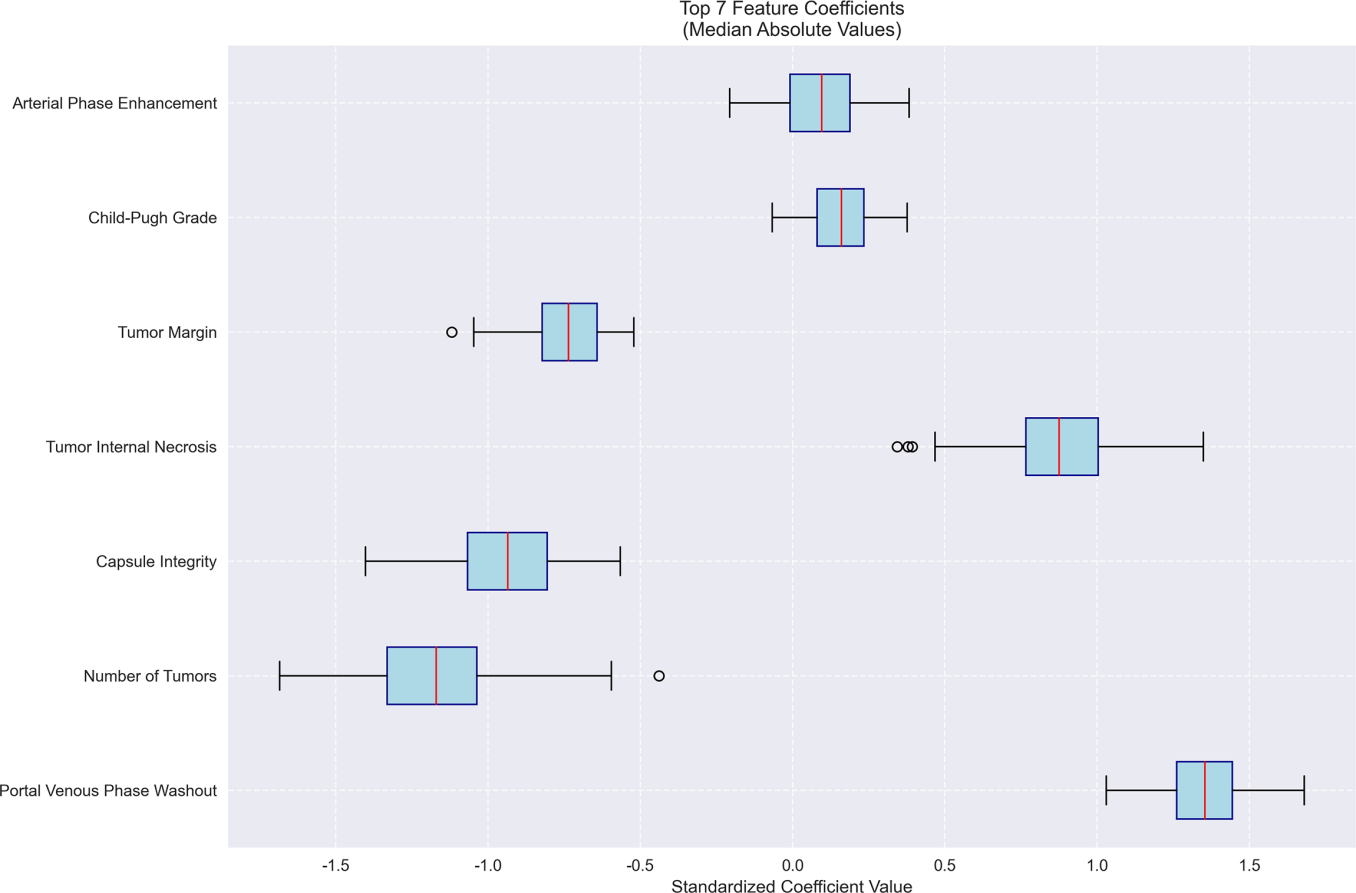
Feature importance for MVI prediction model. Bar plot of standardized coefficient magnitudes for the top 7 features, highlighting arterial phase enhancement and Child-Pugh grade as the most influential predictors.

**Figure 6:**
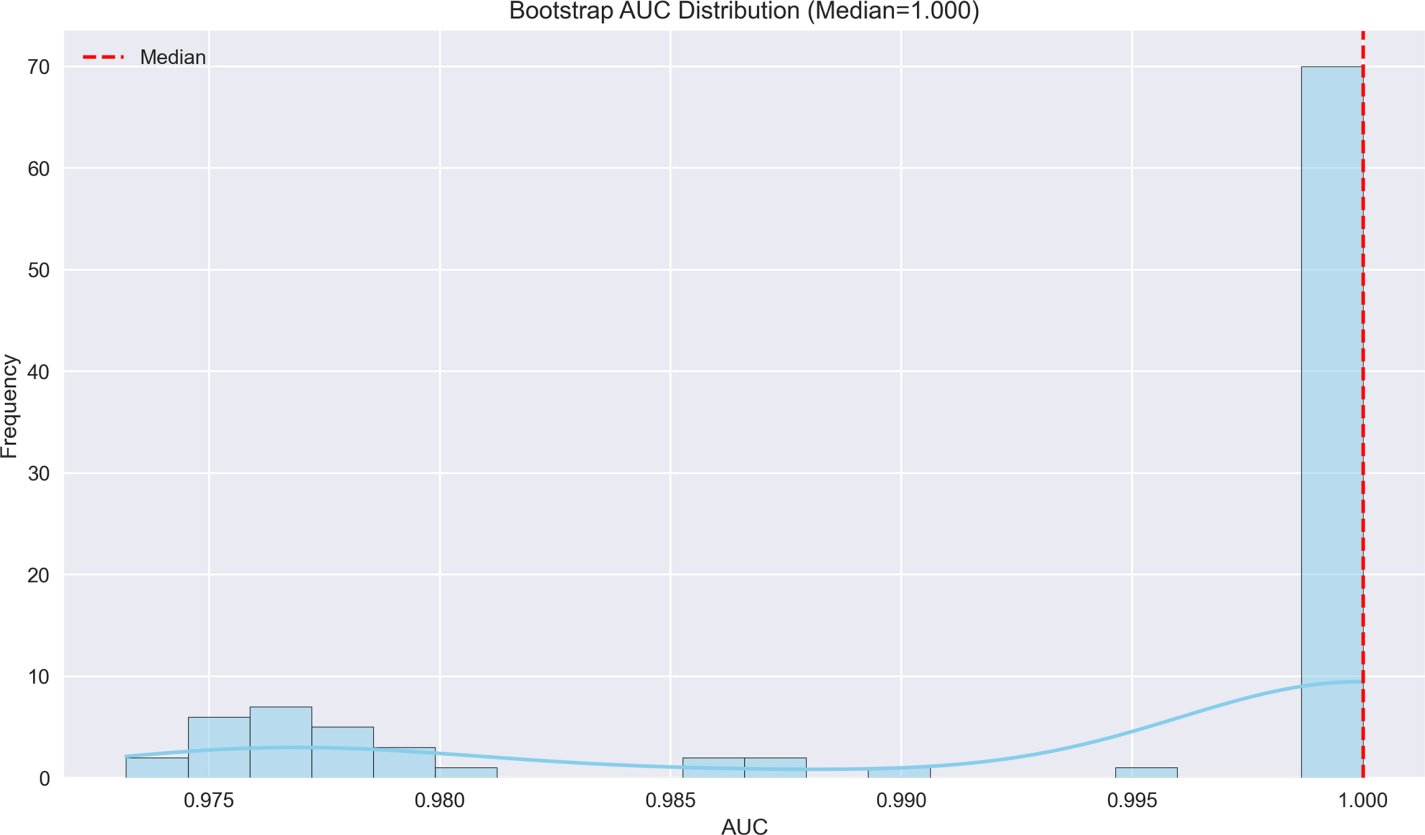
Bootstrap AUC distribution for early recurrence prediction model. The AUC distribution demonstrates exceptional performance (median AUC=1.000), suggesting near-perfect discrimination in predicting early recurrence.

**Figure 7:**
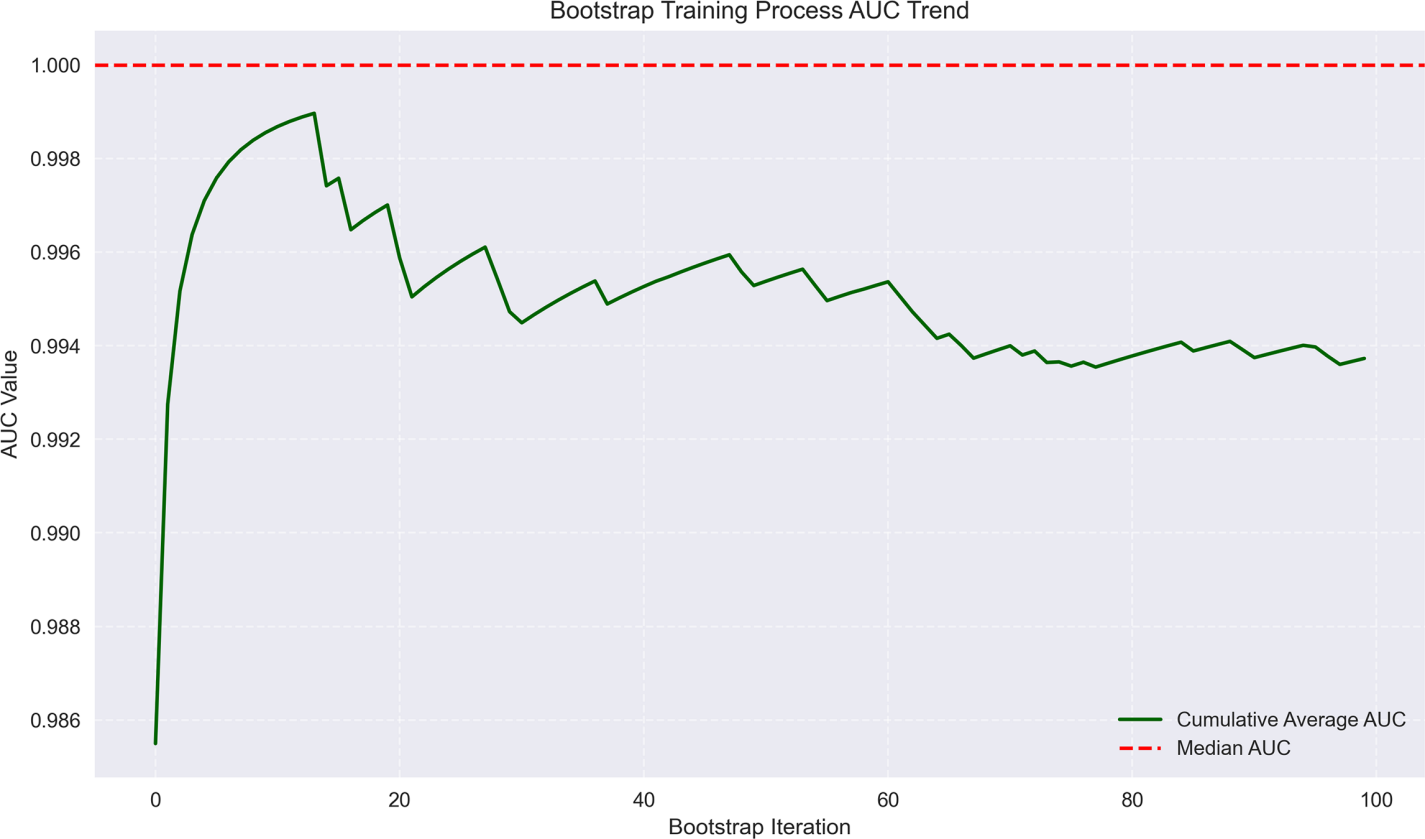
Bootstrap training process AUC trend for early recurrence prediction. The trend line shows AUC values approaching 1.0 across bootstrap iterations, with minimal fluctuation, indicating high model reliability.

**Figure 8:**
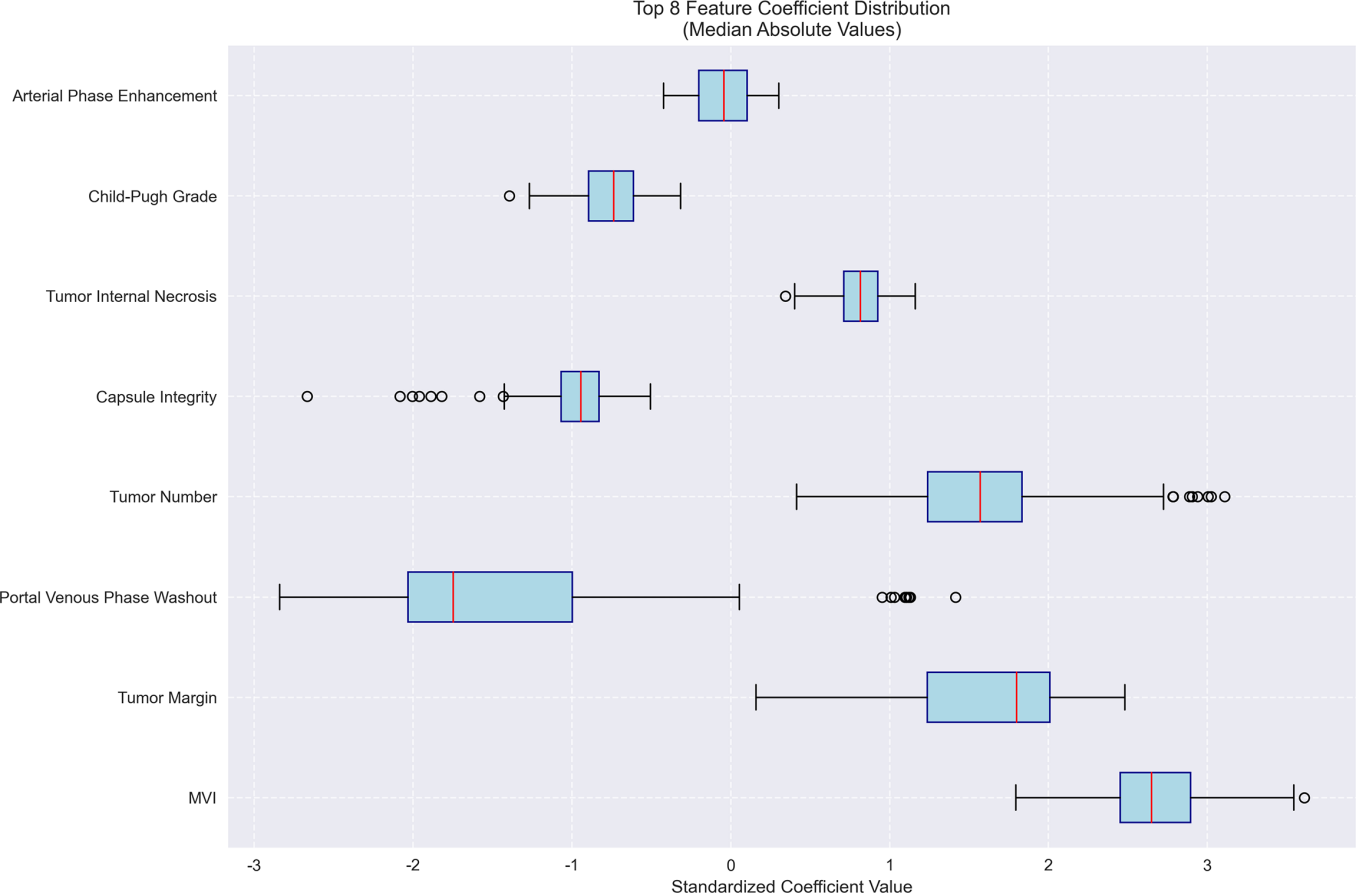
Feature importance for early recurrence prediction model. Bar plot of standardized coefficient magnitudes for the top 7 features, highlighting number of tumors, capsule integrity and tumor margin as the most influential predictors.

We continued to employ a logistic regression model in machine learning to predict the risk of MVI status and HCC recurrence: clinical data were loaded, categorical variables (such as Child-Pugh grade, tumor margin, etc.) were encoded, and numerical variables were standardized with missing values imputed. The dataset was divided into a training dataset and a validation dataset using stratified sampling to ensure that the proportion of outcome events, such as MVI status and early recurrence status, was similar in both sets. The division ratio between the training dataset and validation dataset was 70:30. Bootstrap resampling was performed 100 times to enhance stability, with each iteration involving stratified sampling and feature standardization. A class-balanced Logistic Regression (using the liblinear solver) was applied, and AUC metrics for both training and test sets were calculated. Odds ratios (ORs) with 95% confidence intervals were derived from the median coefficients, and plots including AUC distribution, AUC training trend, feature importance boxplots (**Figures 3**–**8**), OR forest, coefficient distribution violin and correlation heatmaps (**Supplementary Figures 3**–**8**, **Supplementary Tables 3**, **4**) were generated. Key techniques included Bootstrap for small-sample reliability and stratified sampling to maintain class balance. Stratified sampling was performed to maintain class balance, reducing the risk of overestimating the model’s AUC due to an excessively low proportion of outcome events in the validation dataset.

## Results

### Participants’ demographics and baseline characteristics

This study included a total of 186 patients who met the inclusion and exclusion criteria for assessing risk factors for MVI and early recurrence following CT-guided RFA in PRIMARY HCC. Among these patients, 105 were male and 81 were female, reflecting a slight male predominance. Participants’ age ranged from 43 to 76 years, with a mean age of 61.58 ± 5.68 years. All included patients were diagnosed with PRIMARY HCC. Patients were grouped by etiology of cirrhosis, with the majority having HBV-related cirrhosis (45%) and the remaining divided between HCV, NAFLD, and other causes. In terms of liver function, 70% of the patients had Child-Pugh A liver function, and 30% had Child-Pugh B. Tumor characteristics were divided into solitary (58%) and multi-tumor (42%) groups, with the majority of tumors measuring ≤3 cm in diameter (65%). Tumor sizes varied significantly among the cohort, with diameters ranging from 0.51 to 4.96 cm and an average tumor diameter of 2.68 ± 0.86 cm. Of the 186 patients, 58% had a solitary tumor, while 42% had multiple tumors. Among those with multiple tumors, the number of lesions was categorized as follows: 2 lesions (50%), 3 lesions (30%), and ≥4 lesions (20%). Tumor sizes ranged from 0.51 to 4.96 cm, with the distribution of tumor size in solitary tumors and multi-tumor groups as follows: 72% of solitary tumors had a size ≤3 cm, while 60% of multi-tumor cases had tumors ≤3 cm.

### Univariate analysis for predicting MVI in HCC

Potential predictors of MVI following interventional treatment for HCC were systematically evaluated through univariate analysis. Capsule integrity and tumor margin smoothness were significantly associated with MVI. Patients with incomplete capsules or irregular tumor margins had a higher prevalence of MVI, demonstrating that these features may reflect more aggressive tumor biology prone to vascular invasion. The presence of multiple tumors was another strong predictor, and a significant difference was found between patients with solitary versus multiple tumors. Additionally, tumors exhibiting rapid washout in the portal venous phase were more likely to invade microvascular structures. Notably, factors traditionally associated with liver function and tumor biology, such as Child-Pugh grade, tumor internal necrosis, and arterial phase enhancement did not exhibit a statistically significant correlation with MVI in this cohort (**Supplementary Table 1**).

### Identifying risk factors for MVI in HCC using logistic regression analysis

The logistic regression analysis was conducted to identify risk factors for MVI in HCC, and several significant predictors were noted. The presence of multiple tumors significantly increased the risk of MVI, with an OR of 3.348, suggesting that patients with more than one tumor are over three times as likely to experience MVI versus those with a solitary tumor. Incomplete capsules were also a significant predictor, with an OR of 2.498, indicating that tumors lacking a complete capsule are approximately 2.5 times more likely to invade microvascular structures. Irregular tumor margins were strongly associated with MVI, showing an OR of 3.559, highlighting the aggressive nature of tumors with non-smooth margins in promoting vascular invasion. Rapid washout in the portal venous phase was another critical factor, with an OR of 2.982, suggesting an increased likelihood of MVI (**Table 1**). These findings emphasize the value of carefully assessing tumor morphology and dynamics on imaging to identify patients at high risk for MVI. This can play a critical role in developing appropriate therapeutic strategies and predicting prognosis in HCC (**Supplementary Figure 1**; **Figure 1**; **Table 1**).

### Univariate analysis of predictors for early recurrence following intervention in HCC

The univariate analysis identified predictors of early recurrence following interventional treatment for HCC. The analysis examined factors, such as Child-Pugh grade, tumor margin, arterial phase enhancement, portal venous phase washout, tumor internal necrosis, presence of MVI, tumor number, and capsule integrity. Several factors significantly increased the risk of early recurrence. Rapid washout in the portal venous phase was a strong predictor, and the majority of patients in the recurrence group exhibited this feature. Tumor internal necrosis had a significant correlation with recurrence, demonstrating a more aggressive disease phenotype. The presence of MVI was notably higher in patients who experienced recurrence, emphasizing its role as a key prognostic factor. Multiple tumors were also associated with a higher likelihood of early recurrence, reflecting the complexity of more extensive disease. Additionally, incomplete or absent capsule integrity exhibited a significant correlation with recurrence, indicating its potential influence on tumor behavior and treatment outcomes (**Supplementary Table 2**). Other factors, including Child-Pugh grade, tumor margin texture, and arterial phase enhancement were evaluated, while did not reach statistical significance in this analysis.

### Logistic regression analysis identifying predictive factors for early recurrence in HCC post-intervention

The logistic regression analysis assessed various clinical and pathological factors to determine their association with early recurrence following interventional treatment for HCC. Several significant predictors were identified (**Supplementary Figure 2**; **Figure 2**; **Table 1**). Rapid washout in the portal venous phase had a substantial impact on early recurrence, with an OR of 3.213, indicating that patients exhibiting this feature were more than three times as likely to experience recurrence. Tumor internal necrosis was also significantly associated with recurrence, with an OR of 2.376, suggesting that necrotic tumors had a higher tendency for aggressive recurrence. MVI exhibited a strong correlation with early recurrence, with an OR of 3.324, emphasizing its role as a critical prognostic factor. The presence of multiple tumors increased the recurrence risk, with an OR of 2.893, highlighting the influence of a higher tumor burden. Additionally, incomplete capsule integrity was linked to an increased risk of recurrence, with an OR of 3.455, underscoring the importance of capsule integrity in tumor containment and recurrence risk.

### Machine learning of logistic regression model

To enhance the generalizability, predictive performance, credibility, and clinical applicability of our findings, we further conducted machine learning simulations using a logistic regression model. The patients in this study were randomly divided into a training set and a validation set, with bootstrap resampling performed.

### Risk factors for MVI in HCC

The machine learning results demonstrated that portal venous phase washout (present vs. absent) was the strongest risk factor for microvascular invasion (MVI) (OR = 3.87, 95%CI 3.19-4.71, p<0.001), followed by tumor internal necrosis (present vs. absent) (OR = 2.4, 95%CI 1.53-3.24, p<0.001). Although Child-Pugh grade (B vs. A) and arterial phase enhancement (present vs. absent) showed a trend toward increased risk (OR = 1.17 and 1.10, respectively), their 95% confidence intervals included 1 (likely p>0.05), indicating no statistical significance. Among protective factors, solitary tumor (solitary vs. multiple) showed the strongest protective effect (OR = 0.31, 95%CI 0.21-0.50, p<0.001), followed by capsule integrity (intact vs. incomplete) (OR = 0.39, 95%CI 0.28-0.54, p<0.001) and tumor margin regularity (regular vs. irregular) (OR = 0.48, 95%CI 0.37-0.58, p<0.001). These findings suggest that portal venous phase washout and tumor internal necrosis are independent risk factors for MVI, while solitary tumor, intact capsule, and regular margin are significant protective factors. The model demonstrated excellent predictive performance, achieving a median AUC of 0.853 (range: 0.775-0.925) through 100 bootstrap validations. The AUC trend remained stable during training (with the cumulative average AUC ultimately converging between 0.86-0.89), and the optimal single validation AUC reached 0.925, indicating robust discriminative ability and reliability of the model (**Figures 3**–**5**; **Supplementary Figures 3**–**5**; **Supplementary Table 3**).

### Predictive factors for early recurrence in HCC post-intervention

The logistic regression analysis identified several significant predictors for HCC progression, with MVI (present vs absent) demonstrating the strongest association (OR = 14.15, 95%CI 6.16-31.79, p<0.001). Tumor margin regularity (regular vs irregular) showed substantial predictive value (OR = 6.05, 95%CI 1.47-10.49, p=0.002), followed by multiple tumor number (multiple vs solitary) (OR = 4.81, 95%CI 1.64-19.55, p=0.003) and tumor internal necrosis (present vs absent) (OR = 2.26, 95%CI 1.62-2.88, p<0.001). Protective factors included complete capsule integrity (complete vs incomplete) (OR = 0.39, 95%CI 0.14-0.58, p=0.001) and Child-Pugh grade A (vs B) (OR = 0.48, 95%CI 0.31-0.65, p=0.004). Portal venous phase washout (present vs absent) showed borderline significance (OR = 0.17, 95%CI 0.07-3.05, p=0.052), while arterial phase enhancement (present vs absent) was not statistically significant (OR = 0.96, 95%CI 0.71-1.32, p=0.812). These findings highlight MVI as the predominant risk factor while emphasizing the protective role of capsule integrity and preserved liver function in HCC progression. The model demonstrated outstanding predictive performance, achieving a median AUC of 1.000 (range: 0.975–1.000) through 100 bootstrap validations. The AUC trend remained highly stable during training, with the cumulative average AUC consistently above 0.990 and converging near the median value. The model achieved a median AUC of 0.853 (range, 0.775–0.925) for predicting MVI, with stable AUC trends during training and validation phases. However, the median AUC for the early recurrence prediction model was found to be 1.000 (range, 0.975–1.000), which is unusually high and demonstrates potential overfitting. This high value may be attributed to the small sample size and lack of sufficient stratification in the validation dataset. To address this, future studies will explore alternative validation methods or larger datasets to confirm the reliability of the model’s discriminative ability. The optimal single validation AUC reached 1.000, underscoring the model’s exceptional discriminative ability and reliability (**Figures 6**–**8**; **Supplementary Figures 6**–**8**; **Supplementary Table 4**).

## Discussion

The management of primary HCC remains a formidable challenge in oncology, particularly due to the high rates of MVI and early recurrence post-treatment. These phenomena significantly influence patient outcomes and complicate therapeutic strategies. MVI, an indicator of aggressive tumor behavior, involves the spread of cancer cells into the microvasculature surrounding the tumor. It is a critical prognostic factor, as it elevates the risk of both intrahepatic spread and distant metastasis (12, 13). CT-guided RFA is widely adopted for treating early-stage HCC due to its minimally invasive nature and effectiveness in local tumor control. However, its efficacy is markedly reduced when MVI is present, as microscopic tumor deposits may remain post-ablation, increasing the risk of recurrence. Additionally, early recurrence, occurring within two years post-treatment, is another significant challenge, mainly indicating aggressive tumor biology or incomplete initial ablation. This comprehensive analysis of risk factors for MVI and early recurrence following CT-guided RFA in patients with primary HCC has identified several critical biological and morphological predictors (14–16). These predictors include the integrity of the tumor capsule, tumor margin texture, the number of tumors, portal venous phase washout, and tumor internal necrosis. Each factor plays a significant role in tumor biology and can influence the clinical outcomes of interventional treatments.

MVI has been consistently linked to worse outcomes in HCC due to its association with the tumor’s ability to invade and metastasize. The present study indicated that tumors with multiple nodules, incomplete capsules, and irregular margins exhibit higher rates of MVI. These characteristics may signify a more invasive phenotype, potentially due to higher genomic instability or the presence of more aggressive cellular clones in these tumors (17–19). Furthermore, the presence of an incomplete capsule may facilitate tumor cell access to the surrounding vasculature, increasing the likelihood of dissemination. The predictive value of rapid portal venous phase washout for both MVI and early recurrence is linked to its association with higher tumor grade and vascular density (20, 21). Rapid washout often signifies highly vascularized tumors, which are inherently more prone to invading adjacent vessels and metastasizing. This vascular characteristic not only promotes local spread but also serves as a conduit for distant dissemination, particularly within the liver’s highly vascular environment (22, 23). Tumor necrosis, another indicator of poor prognosis, reflects rapid tumor growth outpacing its blood supply, leading to hypoxic areas. These hypoxic regions can drive genetic instability and select for more aggressive cancer cell phenotypes, increasing the likelihood of recurrence and metastasis (24–26). Moreover, the necrotic process might release growth factors and cytokines that promote angiogenesis and tumor cell motility, further contributing to tumor progression and the risk of recurrence.

The comparison between traditional logistic regression and machine learning-enhanced logistic regression reveals both consistencies and nuanced differences in identifying risk factors for MVI and early recurrence in HCC. Both methods consistently identified portal venous phase washout, tumor internal necrosis, multiple tumors, and incomplete capsule integrity as significant risk factors for MVI, with machine learning further refining their predictive strength (e.g., OR = 3.87 vs. 2.982 for washout). Similarly, MVI presence was the strongest predictor of early recurrence in both analyses, though machine learning demonstrated a dramatically higher OR (14.15 vs. 3.324), suggesting enhanced sensitivity in capturing its prognostic impact. Notably, machine learning provided more precise confidence intervals and significance thresholds, clarifying borderline associations (e.g., Child-Pugh grade B and arterial phase enhancement were definitively non-significant). It also highlighted tumor margin regularity as a stronger protective factor (OR = 0.48 vs. 3.559 inverse risk in traditional analysis), underscoring its clinical relevance. The machine learning model’s ability to quantify protective factors (e.g., solitary tumors, intact capsules) with narrower CIs reinforces their utility in risk stratification. These findings suggest that while traditional regression identifies key predictors, machine learning optimizes risk quantification, particularly for high-impact variables like MVI. This advancement supports integrating machine learning into HCC prognostic models to improve individualized therapeutic strategies and surveillance protocols.

While MVI cannot be definitively diagnosed preoperatively, identifying patients with a high likelihood of MVI based on imaging and clinical factors may provide valuable insights for treatment planning. For instance, patients exhibiting characteristics, such as rapid washout in the portal venous phase, tumor internal necrosis, and irregular tumor margins may present with higher risk profiles for MVI, as revealed in this study. Although these factors cannot replace postoperative pathological confirmation, they may help clinicians prioritize surgical resection as a potential treatment option in cases where aggressive tumor biology is suspected. Furthermore, the identification of patients at high risk for MVI could support more personalized, precision-based approaches to treatment selection, ensuring that high-risk patients receive appropriate intervention and monitoring strategies post-procedure.

The presence of multiple tumors is a well-established risk factor for both MVI and early recurrence, potentially reflecting a field cancerization effect in which the liver environment is predisposed to developing multifocal tumors, each with invasive potential. Additionally, multiple tumors may indicate a higher tumor burden and a systemic pro-oncogenic milieu, further increasing the likelihood of recurrence after treatment (27–29). Early recurrence, particularly when associated with factors, such as an incomplete capsule and rapid washout, may also be driven by residual microscopic disease persisting post-ablation. Due to their aggressive nature, these tumors are more likely to harbor satellite lesions and micro-invasions that remain undetectable during initial imaging and treatment, ultimately leading to recurrence (30, 31). The factors identified in this study highlighted the complex correlation among tumor morphology, tumor progression, and the tumor microenvironment in determining outcomes for HCC patients undergoing RFA. These findings highlight the importance of comprehensive preoperative assessment and support an integrated approach, incorporating advanced imaging techniques and molecular profiling to better stratify patients according to risk (32, 33).

To enhance the clinical relevance of the study, the observed risk factors for MVI and early recurrence in patients undergoing CT-guided RFA were compared with those documented in surgical resection cohorts. Several studies have established MVI as a significant predictor of recurrence and poor prognosis following hepatic resection for HCC, with outcomes often reflecting the tumor’s vascular invasion status. Consistent with findings from resection-based studies, the results confirm that factors, such as tumor margin irregularity, capsule integrity, and the presence of multiple tumors contribute to a higher likelihood of MVI and early recurrence. Notably, MVI has been identified as a primary risk factor for recurrence in resected HCC specimens, highlighting the clinical importance of early identification and intervention in patients at high risk for MVI, regardless of the treatment modality (resection or ablation). In particular, the predictive value of imaging features, such as portal venous phase washout, aligns with surgical outcomes, where vascular invasion often correlates with poor long-term survival following resection. Additionally, the findings support the growing emphasis on personalized treatment strategies, where the presence of MVI and tumor characteristics guide therapeutic decision-making for both RFA and resection cohorts.

## Limitations

This study, while being comprehensive, presents several limitations that should be addressed in the future research. Firstly, the retrospective design of the study might introduce selection biases and limit the ability to establish causality. Additionally, as the study was conducted at a single institution, the generalizability of the findings to broader populations might be limited. While the sample size was sufficient, expanding it in future studies may enhance statistical power and allow for more detailed subgroup analyses. Hence, prospective multicenter studies are necessary to validate these findings and mitigate the biases inherent in retrospective research. Further investigation should also incorporate advanced imaging techniques and molecular profiling to gain deeper insights into the biological progress driving MVI and early recurrence. This could lead to the development of predictive models and more personalized therapeutic strategies. Additionally, investigating the impact of combining RFA with other modalities, such as targeted therapy or immunotherapy, could provide insights into improving outcomes for patients with HCC who are at high risk of recurrence and invasion. The absence of detailed baseline characteristics, particularly the distribution of etiologies and liver function status, might limit our ability to accurately stratify risk in subgroups of patients with different cirrhosis etiologies. Future studies should incorporate these variables to better predict MVI and recurrence risk based on cirrhosis etiology, tumor number, and liver function status. The observed association between tumor number and MVI risk supports the need for more granular data on tumor characteristics, improving clinical decision-making for early intervention.

## Conclusions

Patients with HCC exhibiting multiple tumors, incomplete capsules, irregular tumor margins, and rapid portal venous washout face an increasing risk of MVI following surgery. Additionally, rapid portal venous phase washout, tumor internal necrosis, MVI, multiple tumors, and incomplete capsule may increase the likelihood of early recurrence following treatment. These findings suggest the importance of targeted monitoring and personalized therapeutic strategies for high-risk patients.

## Data Availability

The original contributions presented in the study are included in the article/**Supplementary Material**. Further inquiries can be directed to the corresponding author.
