# Supplementary figures and images for: Microvascular invasion and early recurrence of hepatocellular carcinoma after CT-guided radiofrequency ablation: risk factor analysis
Source: Front Oncol. 2025 Oct 21;15:1672300. doi: 10.3389/fonc.2025.1672300 (PMC12583091; doi:10.3389/fonc.2025.1672300)

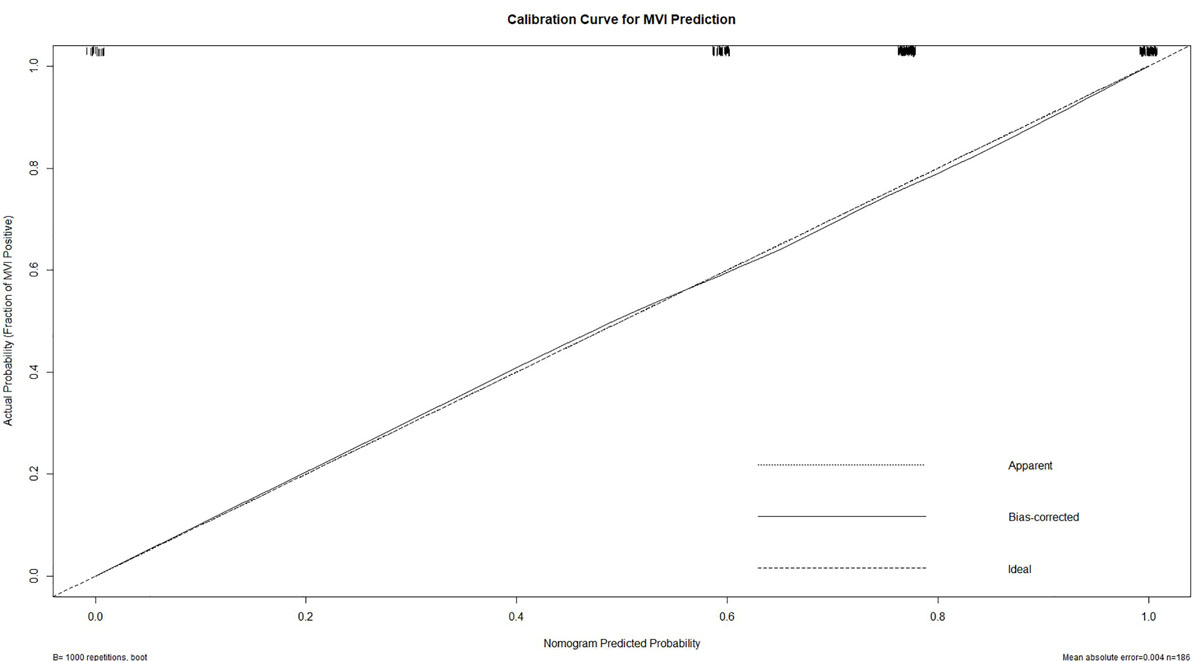

Supplement: Supplementary file 1 [file Image1.jpeg]

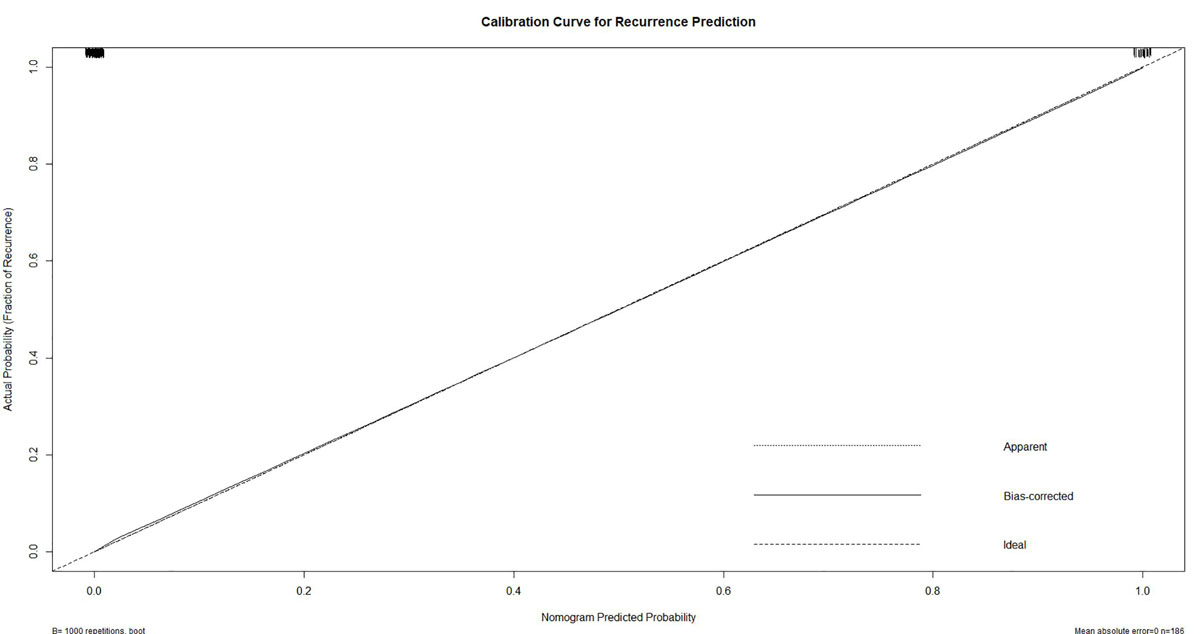

Supplement: Supplementary file 2 [file Image2.jpeg]

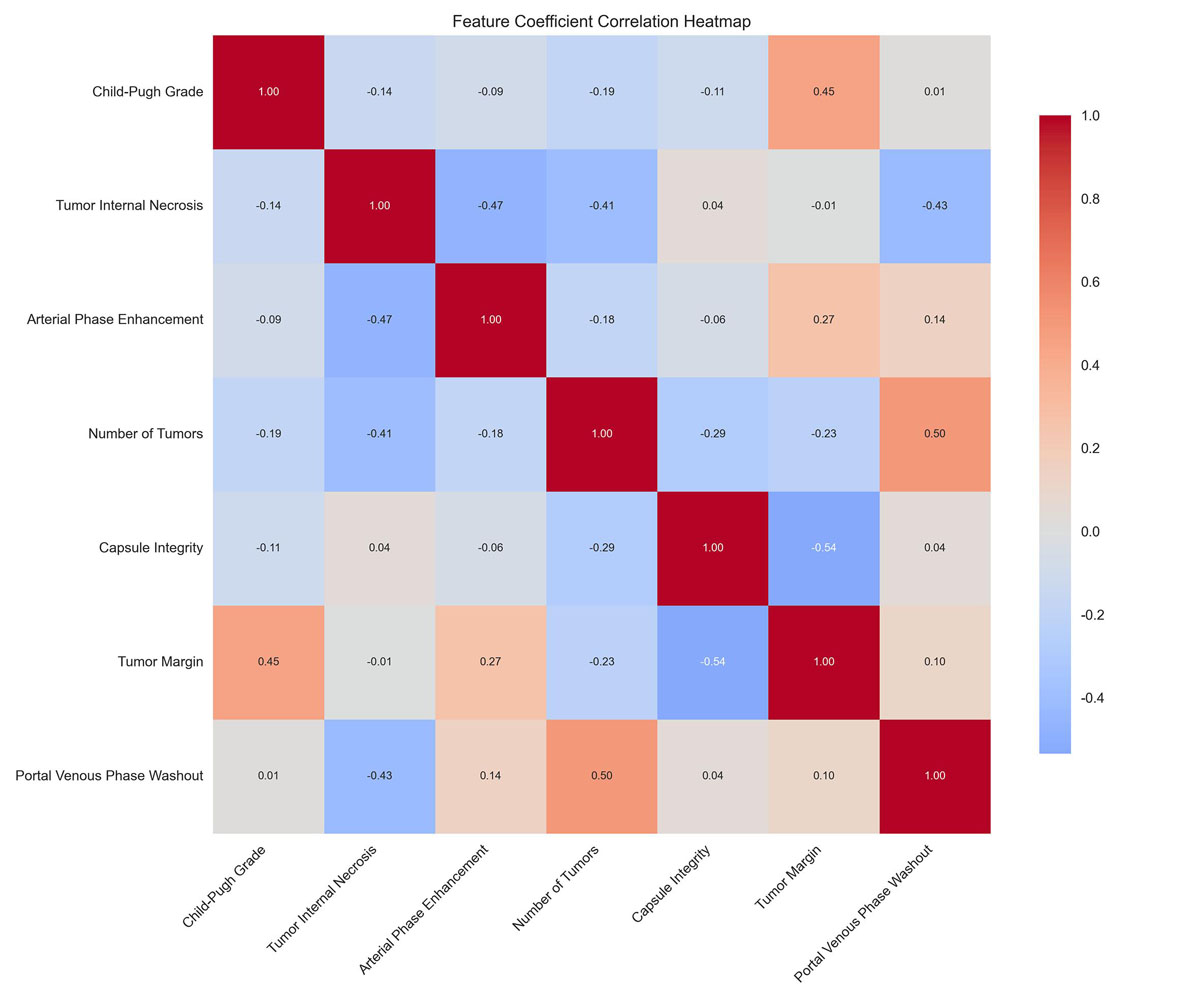

Supplement: Supplementary file 3 [file Image3.jpeg]

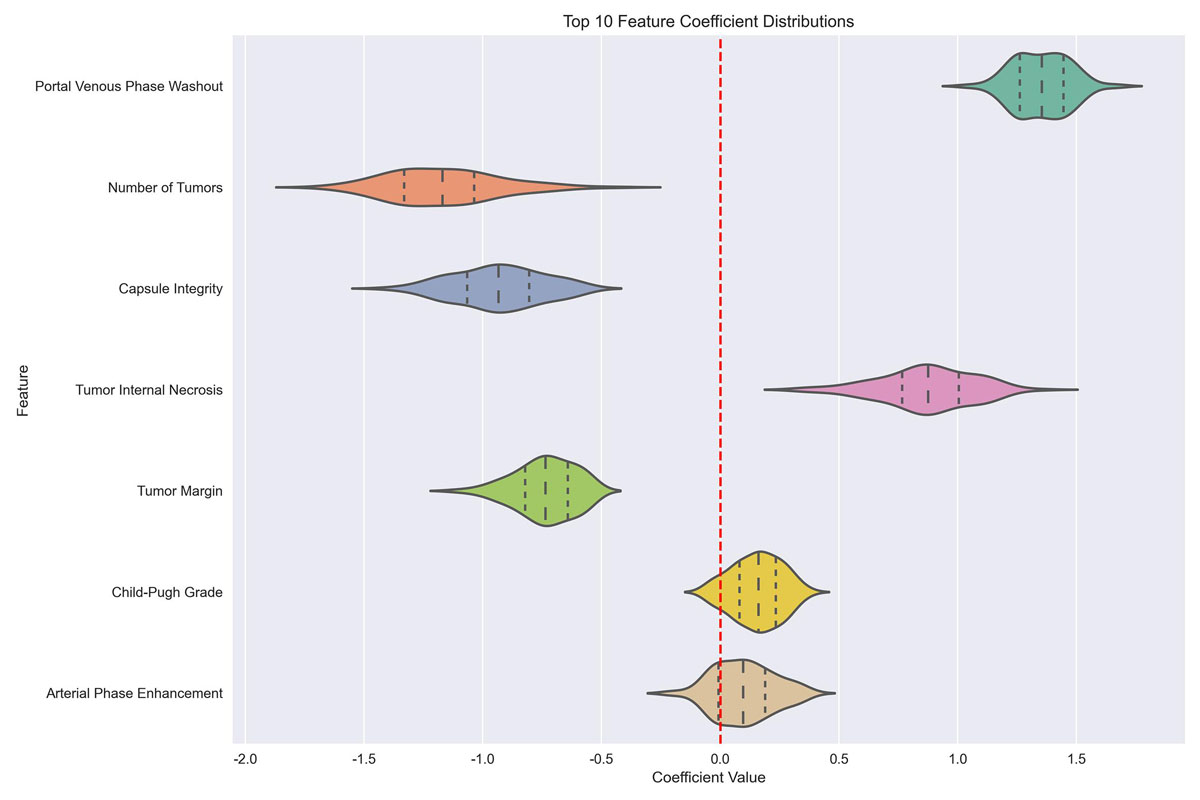

Supplement: Supplementary file 4 [file Image4.jpeg]

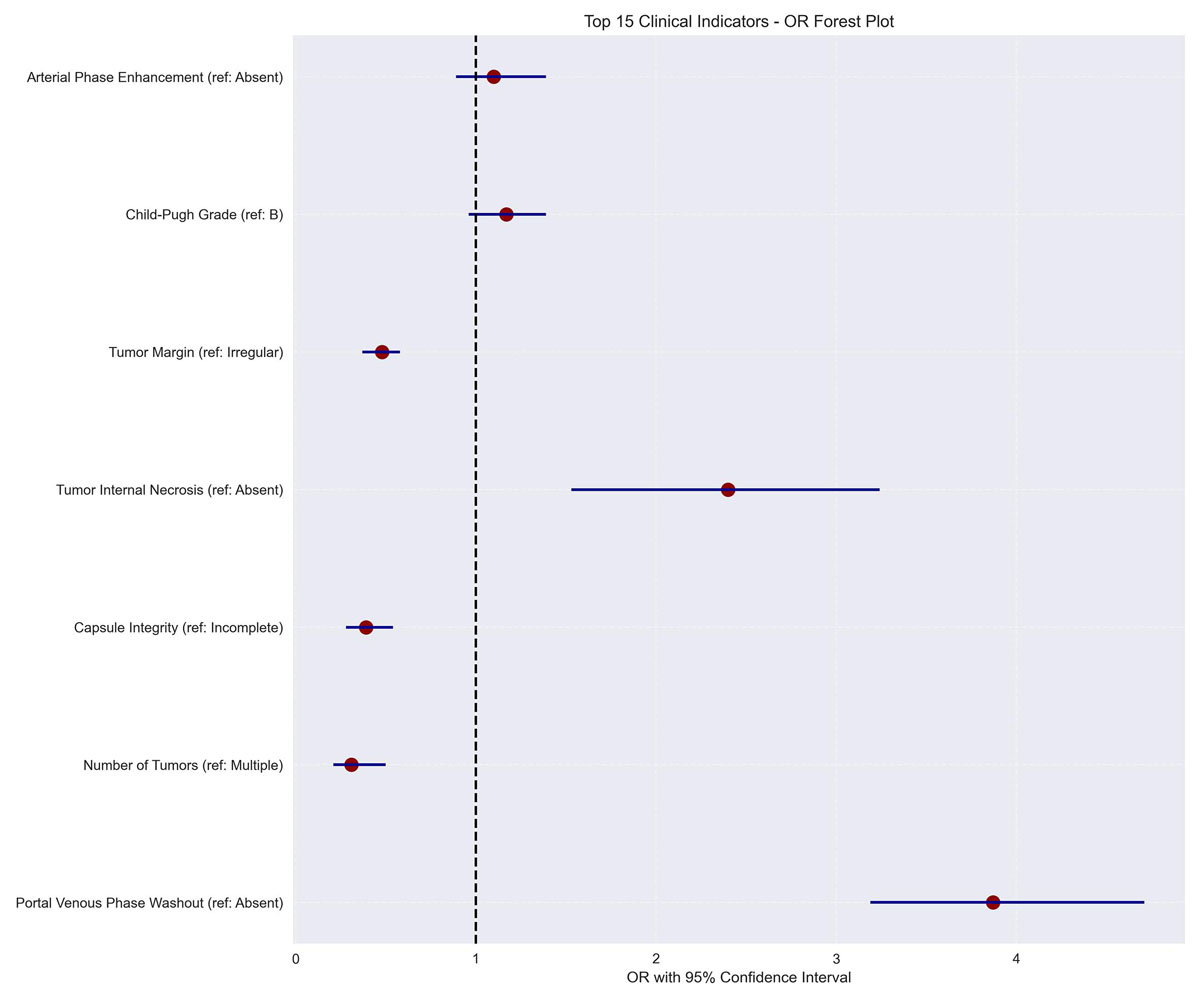

Supplement: Supplementary file 5 [file Image5.jpeg]

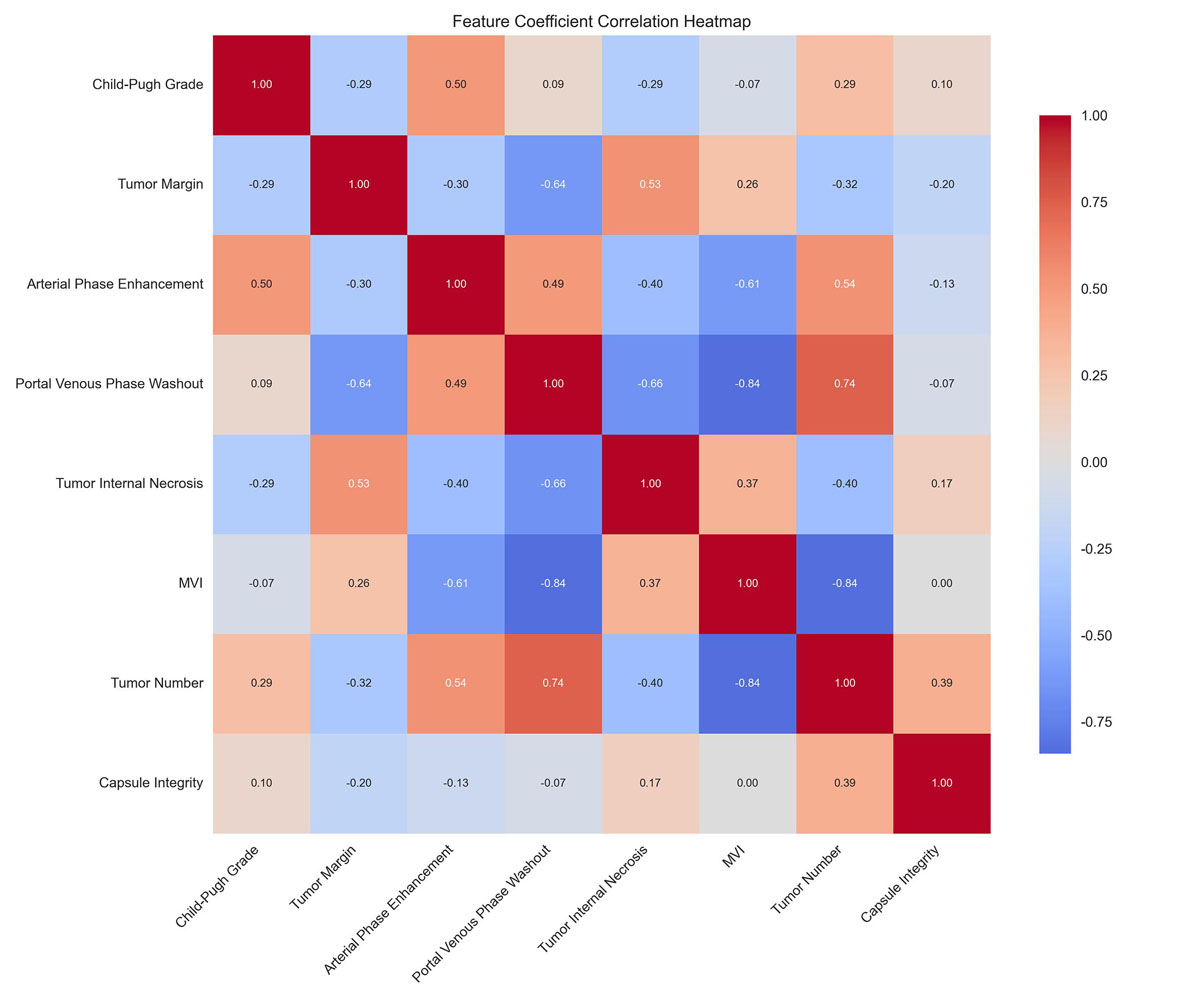

Supplement: Supplementary file 6 [file Image6.jpeg]

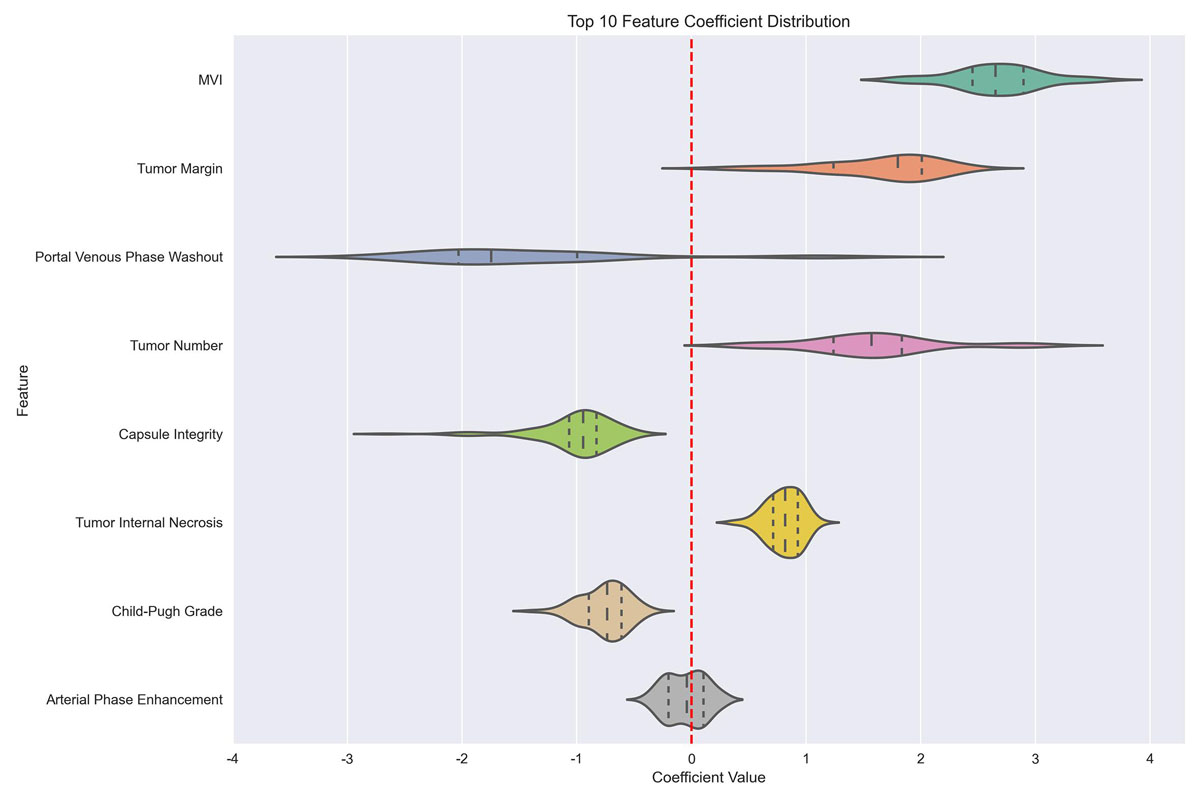

Supplement: Supplementary file 7 [file Image7.jpeg]

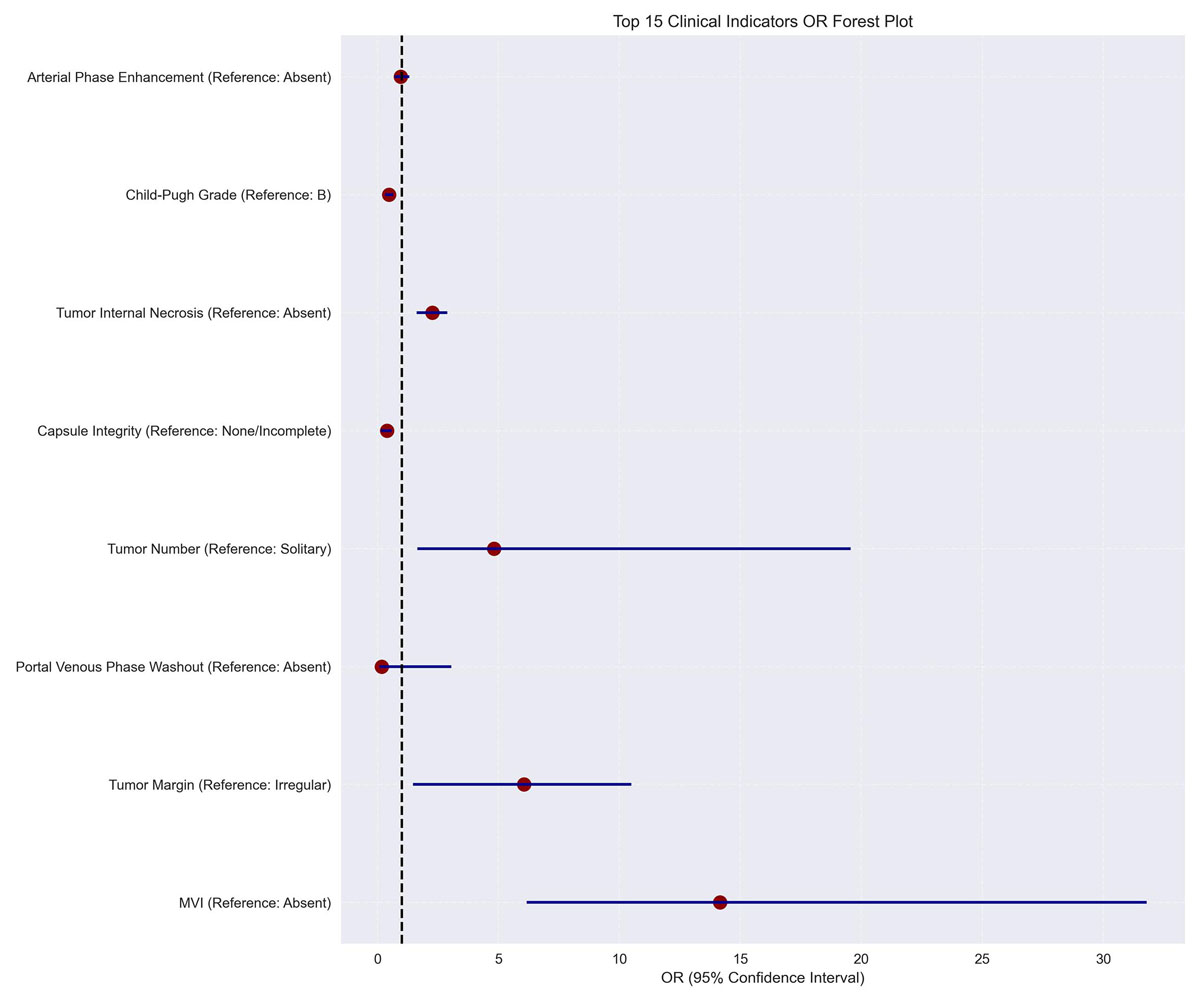

Supplement: Supplementary file 8 [file Image8.jpeg]
