# Supplementary material for: Microvascular invasion and early recurrence of hepatocellular carcinoma after CT-guided radiofrequency ablation: risk factor analysis
Source: Front Oncol. 2025 Oct 21;15:1672300. doi: 10.3389/fonc.2025.1672300 (PMC12583091; doi:10.3389/fonc.2025.1672300)
Supplement: Supplementary file 9 [file Table1.docx]

Supplementary Table 1. Univariate Analysis of Risk Factors for Microvascular Invasion (MVI) Following Interventional Treatment of Hepatocellular Carcinoma.

| Risk factor | Group | MVI positive (n=136) | Non-MVI (n=50) | χ² | p-value |
| --- | --- | --- | --- | --- | --- |
| Child-Pugh Grade | A | 59 (43.38%) | 30 (60%) | 0.83 | 0.36 |
|  | B | 77 (56.62%) | 20 (40%) |  |  |
| Tumor Internal Necrosis | Present | 40 (29.41%) | 15 (30%) | 1.42 | 0.233 |
|  | Absent | 96 (70.59%) | 35 (70%) |  |  |
| Arterial Phase Enhancement | Present | 46 (33.82%) | 22 (44%) | 0.11 | 0.74 |
|  | Absent | 90 (66.18%) | 28 (56%) |  |  |
| Number of Tumors | Solitary | 31 (22.79%) | 33 (66%) | 8.15 | <0.01 |
|  | Multiple | 105 (77.21%) | 17 (34%) |  |  |
| Capsule Integrity | Intact | 22 (16.18%) | 28 (56%) | 13.9 | <0.01 |
|  | Incomplete | 114 (83.82%) | 22 (44%) |  |  |
| Tumor Margin | Smooth | 25 (18.38%) | 30 (60%) | 10.2 | <0.01 |
|  | Irregular | 111 (81.62%) | 20 (40%) |  |  |
| Portal Venous Phase Washout | Present | 79 (58.09%) | 18 (36%) | 6.95 | <0.01 |
|  | Absent | 57 (41.91%) | 32 (64%) |  |  |
