# Supplementary material for: Microvascular invasion and early recurrence of hepatocellular carcinoma after CT-guided radiofrequency ablation: risk factor analysis
Source: Front Oncol. 2025 Oct 21;15:1672300. doi: 10.3389/fonc.2025.1672300 (PMC12583091; doi:10.3389/fonc.2025.1672300)
Supplement: Supplementary file 10 [file Table2.docx]

Supplementary Table 2. Univariate Analysis of Predictors for Early Recurrence Post-Intervention in Hepatocellular Carcinoma.

| Risk factor | Recurrence group (n=33) | Non-recurrence group (n=153) | χ²/t | p-value |
| --- | --- | --- | --- | --- |
| Child-Pugh Grade |  |  | 3.7 | 0.054 |
| - A | 6 (18.18%) | 53 (34.64%) |  |  |
| - B | 27 (81.82%) | 100 (65.36%) |  |  |
| Tumor Margin |  |  | 2.4 | 0.121 |
| - Smooth | 10 (30.30%) | 51 (33.33%) |  |  |
| - Irregular | 23 (69.70%) | 102 (66.67%) |  |  |
| Arterial Phase Enhancement |  |  | 0.05 | 0.823 |
| - Present | 15 (45.45%) | 58 (37.91%) |  |  |
| - Absent | 18 (54.55%) | 95 (62.09%) |  |  |
| Portal Venous Phase Washout |  |  | 4.2 | <0.05 |
| - Present | 30 (90.91%) | 51 (33.33%) |  |  |
| - Absent | 3 (9.09%) | 102 (66.67%) |  |  |
| Tumor Internal Necrosis |  |  | 3.97 | <0.05 |
| - Present | 15 (45.45%) | 28 (18.30%) |  |  |
| - Absent | 18 (54.55%) | 125 (81.70%) |  |  |
| MVI |  |  | 4.6 | <0.05 |
| - Present | 28 (84.85%) | 46 (30.07%) |  |  |
| - Absent | 5 (15.15%) | 107 (69.93%) |  |  |
| Tumor Number |  |  | 7.37 | <0.05 |
| - Solitary | 3 (9.09%) | 87 (56.86%) |  |  |
| - Multiple | 30 (90.91%) | 66 (43.14%) |  |  |
| Capsule Integrity |  |  | 8.64 | <0.05 |
| - Intact | 5 (15.15%) | 45 (49.02%) |  |  |
| - None/Incomplete | 28 (84.85%) | 108 (50.98%) |  |  |
