# Supplementary material for: Microvascular invasion and early recurrence of hepatocellular carcinoma after CT-guided radiofrequency ablation: risk factor analysis
Source: Front Oncol. 2025 Oct 21;15:1672300. doi: 10.3389/fonc.2025.1672300 (PMC12583091; doi:10.3389/fonc.2025.1672300)
Supplement: Supplementary file 11 [file Table3.docx]

Supplementary Table 3 OR_analysis for MVI.

| Variable | OR | 95% CI |
| --- | --- | --- |
| Portal Venous Phase Washout (ref: Absent) | 3.87 | [3.19, 4.71] |
| Number of Tumors (ref: Multiple) | 0.31 | [0.21, 0.50] |
| Capsule Integrity (ref: Incomplete) | 0.39 | [0.28, 0.54] |
| Tumor Internal Necrosis (ref: Absent) | 2.4 | [1.53, 3.24] |
| Tumor Margin (ref: Irregular) | 0.48 | [0.37, 0.58] |
| Child-Pugh Grade (ref: B) | 1.17 | [0.96, 1.39] |
| Arterial Phase Enhancement (ref: Absent) | 1.1 | [0.89, 1.39] |
| Portal Venous Phase Washout (ref: Absent) | 3.87 | [3.19, 4.71] |
