# Supplementary material for: Microvascular invasion and early recurrence of hepatocellular carcinoma after CT-guided radiofrequency ablation: risk factor analysis
Source: Front Oncol. 2025 Oct 21;15:1672300. doi: 10.3389/fonc.2025.1672300 (PMC12583091; doi:10.3389/fonc.2025.1672300)
Supplement: Supplementary file 12 [file Table4.docx]

Supplementary Table 4 OR_analysis for early recurrence.

| Variable | OR | 95% CI |
| --- | --- | --- |
| MVI (Reference: Absent) | 14.15 | [6.16, 31.79] |
| Tumor Margin (Reference: Irregular) | 6.05 | [1.47, 10.49] |
| Portal Venous Phase Washout (Reference: Absent) | 0.17 | [0.07, 3.05] |
| Tumor Number (Reference: Solitary) | 4.81 | [1.64, 19.55] |
| Capsule Integrity (Reference: None/Incomplete) | 0.39 | [0.14, 0.58] |
| Tumor Internal Necrosis (Reference: Absent) | 2.26 | [1.62, 2.88] |
| Child-Pugh Grade (Reference: B) | 0.48 | [0.31, 0.65] |
| Arterial Phase Enhancement (Reference: Absent) | 0.96 | [0.71, 1.32] |
